# Post-Transcriptional Regulation of 5-Lipoxygenase mRNA Expression via Alternative Splicing and Nonsense-Mediated mRNA Decay

**DOI:** 10.1371/journal.pone.0031363

**Published:** 2012-02-21

**Authors:** Meike J. Ochs, Bernd L. Sorg, Laura Pufahl, Manuel Grez, Beatrix Suess, Dieter Steinhilber

**Affiliations:** 1 Institute of Pharmaceutical Chemistry, Goethe University Frankfurt, Frankfurt, Germany; 2 Institute of Molecular Biosciences, Goethe University Frankfurt, Frankfurt, Germany; 3 Georg-Speyer-Haus, Frankfurt, Germany; Instituto de Biofisica Carlos Chagas Filho, Universidade Federal do Rio de Janeiro, Brazil

## Abstract

5-Lipoxygenase (5-LO) catalyzes the two initial steps in the biosynthesis of leukotrienes (LT), a group of inflammatory lipid mediators derived from arachidonic acid. Here, we investigated the regulation of 5-LO mRNA expression by alternative splicing and nonsense-mediated mRNA decay (NMD). In the present study, we report the identification of 2 truncated transcripts and 4 novel 5-LO splice variants containing premature termination codons (PTC). The characterization of one of the splice variants, 5-LOΔ3, revealed that it is a target for NMD since knockdown of the NMD factors UPF1, UPF2 and UPF3b in the human monocytic cell line Mono Mac 6 (MM6) altered the expression of 5-LOΔ3 mRNA up to 2-fold in a cell differentiation-dependent manner suggesting that cell differentiation alters the composition or function of the NMD complex. In contrast, the mature 5-LO mRNA transcript was not affected by UPF knockdown. Thus, the data suggest that the coupling of alternative splicing and NMD is involved in the regulation of 5-LO gene expression.

## Introduction

5-Lipoxygenase (arachidonate∶oxygen 5-oxido-reductase) is the key enzyme in the biosynthesis of LTs which represent mediators of inflammatory and allergic reactions that are synthesized and released after cell stimulation [1], [2]. Several studies have shown that 5-LO and LTs play a central role in asthma and inflammatory disorders, vascular diseases and cancer [3]. 5-LO is mainly expressed in mature leukocytes which is in agreement with the function of LTs as mediators of immune reactions. Granulocytes, monocytes/macrophages, mast cells, dendritic cells and B-lymphocytes express 5-LO whereas platelets, endothelial cells, T-cells and erythrocytes are 5-LO negative. In the skin, Langerhans cells strongly express 5-LO [4]. During myeloid cell maturation, expression of 5-LO mRNA and protein is strongly upregulated by calcitriol and transforming growth factor-β (TGFβ) in a promoter-independent manner [5], [6], [7].

The existence of 5-LO splice variants was first described by Black and co-workers in human brain tumors [8]. Using Northern blot analysis they have shown that the 5-LO gene is expressed as a multi-transcript family (2.7, 3.1, 6.4 and 8.6 kb) in human brain tumors and in dimethyl sulfoxide-differentiated HL-60 cells. Furthermore, the abundance of 5-LO transcripts was correlated with the malignancy of the brain tumors. Accordingly, it was hypothesized that the 5-LO splice variants play a role in human tumor induced brain edemas. A recent study has investigated whether splice variants of 5-LO exist in human leukocytes. Boudreau *et al.* described two novel 5-LO protein isoforms in human leukocytes that are generated from transcripts lacking exon 13 and parts of exon 10, respectively. Both isoforms are catalytically inactive and inhibit the biosynthetic capacity of wild type (WT) 5-LO in cells. It was suggested that these 5-LO isomers might act as endogenous 5-LO inhibitor that could be involved in the regulation of LT biosynthesis *in vivo* [9].

Alternative splicing is responsible for the generation of multiple transcripts from a single gene. It has been shown that about half of the genes of the human genome are alternatively spliced whereas RNA arrays and sequencing data indicated that even up to 94% of all human genes might be alternatively spliced [10], [11]. About one third of all alternatively spliced genes result in nonsense transcripts containing PTCs. These splice variants can be subsequently subjected to NMD which is a surveillance mechanism that selectively degrades nonsense transcripts [12], [13]. Upon demand, a change in the splicing pattern will immediately result in the mature transcript. Thus, gene expression can be regulated post-transcriptionally by generating alternatively spliced transcripts which are degraded by NMD but can rapidly switch to productive splicing by changing the splicing pattern. The coupling of alternative splicing and NMD seems to represent a widely occurring mechanism by which gene expression is controlled [13]. Brenner and co-workers have termed this process regulated unproductive splicing and translation [12], [13], [14].

In the present study, we have identified novel splice variants of 5-LO in TGFβ/calcitriol differentiated and undifferentiated MM6 cells. Studies with the protein biosynthesis inhibitor puromycin and knockdown of individual NMD factors revealed that the splice variant 5-LOΔ3, but not the WT 5-LO mRNA is regulated by NMD in a UPF-dependent manner. Interestingly, the effects of the knockdown of UPF1 and UPF3b on 5-LOΔ3 mRNA expression are dependent on the differentiation status of the cells suggesting that cell differentiation alters the composition or function of the NMD complex.

## Results

### Identification and characterization of novel 5-LO splice variants in TGFβ and calcitriol differentiated and undifferentiated MM6 cells

**Figure 1:**
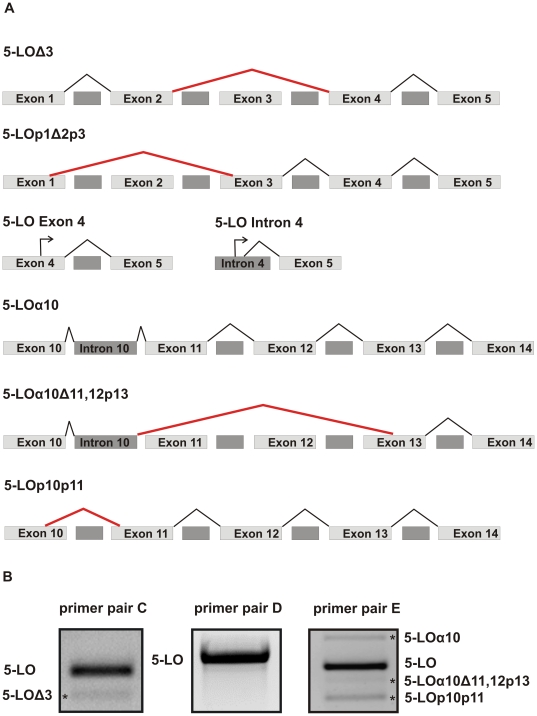
Identification of novel 5-LO splice variants in MM6 cells. (A) Schematic representation of the identified 5-LO splice variants. (B) RT-PCR products obtained with primer pair C (exon 2 to exon 5), D (exon 5 to exon 10) and E (exon 10 to exon 14) separated on a 1.5% agarose gel. Asterisks indicate the alternative splicing products whose existence was confirmed by at least one sequenced recombinant clone.

To investigate the existence of 5-LO splice variants, rapid amplification of 5′ cDNA ends (5′ RACE) and reverse transcription polymerase chain reaction (RT-PCR) was performed with RNA isolated from undifferentiated and TGFβ/calcitriol differentiated MM6 cells. 5′RACE revealed the presence of four 5-LO splice variants (Fig. 1A). The most abundant variant 5-LOΔ3 completely lacks exon 3. In a second splice variant, the end of the exon 1, the complete exon 2 and the beginning of exon 3 (5-LOp1Δ2p3) was deleted. In addition, we identified two transcripts with an alternative transcription initiation site within exon 4 (5-LO Exon 4) and intron 4 (5-LO Intron 4), respectively.

RT-PCR was carried out to confirm the results from the 5′ RACE and to detect 5-LO splice variants in a more downstream region of the gene. To this aim, eight primer pairs (A–G, Fig. S1) were designed which cover the complete 5-LO gene. The analysis of the first five exons confirmed the existence of 5-LOΔ3 (Fig. 1B). RT-PCR revealed further alternatively spliced transcripts in the 3′ part of the 5-LO gene, one with an intron 10 retention (5-LOα10) as already described [9], one with an intron 10 retention and deletion of exons 11, exon 12 and the beginning of exon 13 (5-LOα10Δ11,12p13) and another splice variant lacking parts of exon 10 and exon 11 (5-LOp10p11, Fig. 1B). No alternative splicing was detected in the central part of the 5-LO transcript using primer pair D (Fig. 1B). Figure 1A gives an overview on 5-LO splice variants found via 5′RACE and RT-PCR in MM6 cells.

### Expression analysis of alternatively spliced 5-LO transcripts in undifferentiated and TGFβ/calcitriol differentiated MM6 cells

**Figure 2:**
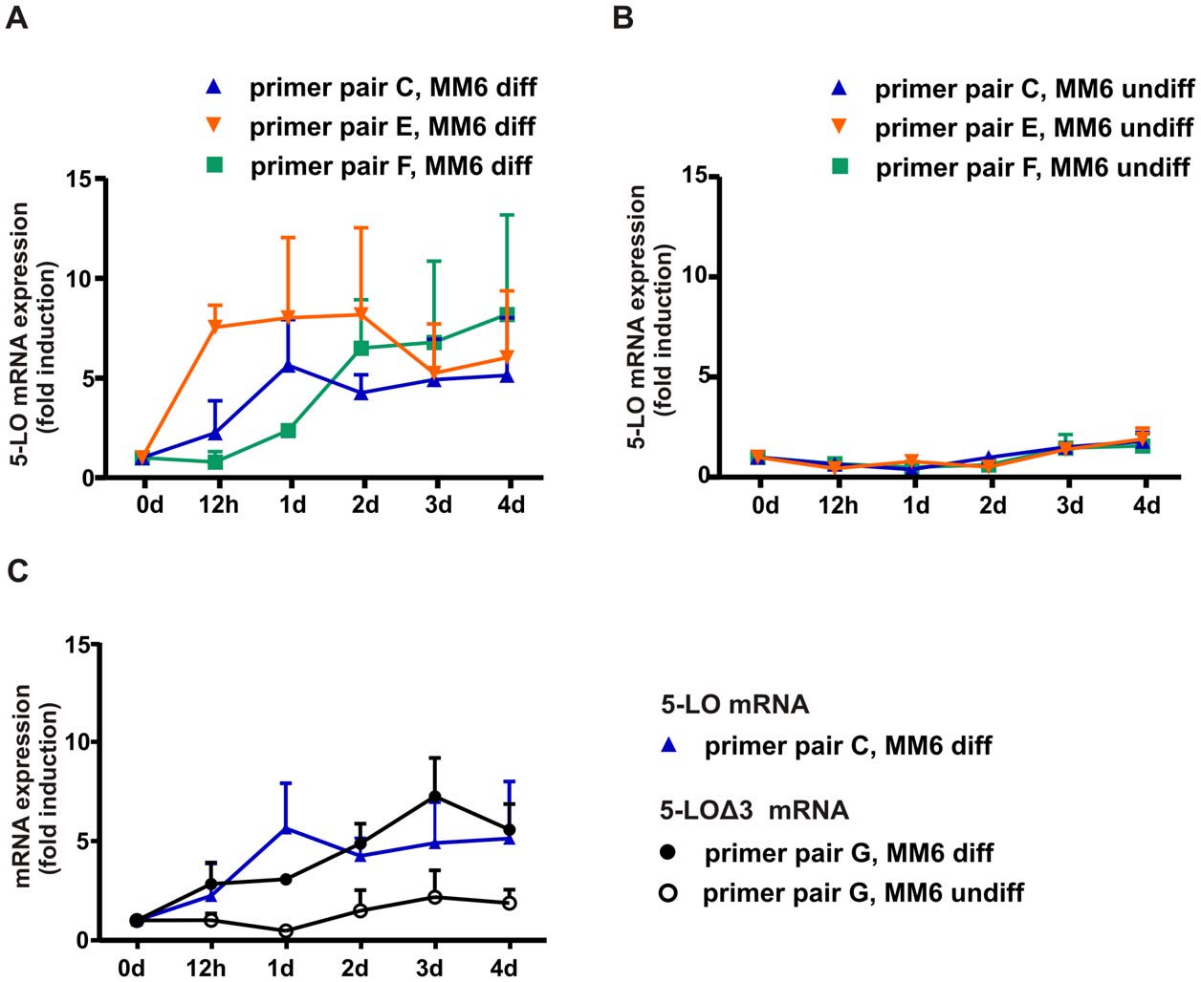
Quantification of alternatively and correctly spliced 5-LO mRNA. Time courses of 5-LO mRNA induction in MM6 cells cultured with (diff) and without (undiff) TGFβ (1 ng/ml) and calcitriol (50 nM). Total RNA was extracted and cDNA was prepared using random hexamer primers for the PCR analysis of correctly spliced 5-LO pre-mRNA applying primer pair C (exon 2 to exon 5) and E (exon 10 to exon 14). 5-LOΔ3 mRNA was determined by primer pair G. For the specific analysis of the mature 5-LO transcripts, the samples were reverse transcribed with oligo-dT priming and the cDNA was amplified with primer pair F (exon 13 to 3′UTR). β-actin mRNA served as constitutively expressed control. The relative changes to day 0 are given as the mean+SE of three independent experiments. Time courses of 5-LO mRNA in (A) differentiated and (B) undifferentiated MM6 cells. (C) Time courses of 5-LOΔ3 mRNA in MM6 cells cultured with (diff) and without (undiff) TGFβ (1 ng/ml) and calcitriol (50 nM). 5-LOΔ3 cDNA was analyzed using primer pair G. For the analysis of correctly spliced 5-LO mRNA the primer pair C was used.

The expression level of different splice variants was determined by RT-PCR analysis. For this purpose MM6 cells were cultured in the absence and presence of TGFβ and calcitriol for 4 days. RNA was isolated at different time points (0 h, 12 h, 1 d, 2 d, 3 d and 4 d) and analyzed for 5-LO mRNA by RT-PCR. In order to detect the various 5-LO transcripts, the RNA was reverse transcribed using random hexamer primers and PCR was carried out with the primer pair C (exon 2 to exon 5) and E (exon 10 to exon 14) to detect mature 5-LO transcripts and with the primer pair G which allows detection of 5-LOΔ3 mRNA (Fig. S1). Polyadenylated and spliced 5-LO mRNA was analyzed using reverse transcription with oligo-dT priming and PCR with primer pair F (exon 13 to 3′UTR). The results displayed a strong induction of 5-LO mRNA expression by TGFβ and calcitriol as previously described [6]. The time course of the induction of 5-LO mRNA strongly depends on which part of the pre-mRNA is analyzed (Fig. 2). For the proximal part of the transcript (exon 2 to exon 5), the most prominent effect was obtained after 1 d whereas the distal part (exon 10 to exon 14) was induced ∼8.0-fold already after 12 h. After 3 d a similar induction was observed with all analyzed 5-LO mRNA species (∼5.0-fold). Interestingly, the mature (spliced and polyadenylated) 5-LO transcripts show a delay in the induction and require 2 d of treatment with TGFβ/calcitriol for complete upregulation (Fig. 2A). In undifferentiated MM6 cells no significant induction of correctly spliced 5-LO mRNA was observed in the distal as well as the proximal part (Fig. 2B).

Next, the splice variant 5-LOΔ3 was analyzed. It shows a similar time course as the correctly spliced transcript (exon 2 to exon 5) which suggests that cell differentiation by TGFβ and calcitriol does not affect splicing of exon 3 (Fig. 2C). Taken together, the data show a rather rapid and strong upregulation of correctly as well as alternatively spliced 5-LO primary transcripts with PTC and a delay in the appearance of the mature (polyadenylated) transcript.

### NMD analysis of 5-LOΔ3 mRNA in undifferentiated and TGFβ/calcitriol differentiated MM6 cells

Since the upregulation of alternatively spliced 5-LO mRNAs with PTCs preceded the induction of mature transcripts, we wondered whether these mRNAs are degraded by NMD and subsequently not subjected to the maturation process. Thus, we investigated whether the most abundant alternatively spliced transcript, 5-LOΔ3 is a substrate for NMD. This 5-LO splice variant shows a frame shift in exon 4 which introduces a stop codon into this exon. The stop codon is located more than 55 nucleotides upstream of the next exon-exon junction which suggests that it represents a PTC [15] so that 5-LOΔ3 mRNA could be a target of NMD.

**Figure 3:**
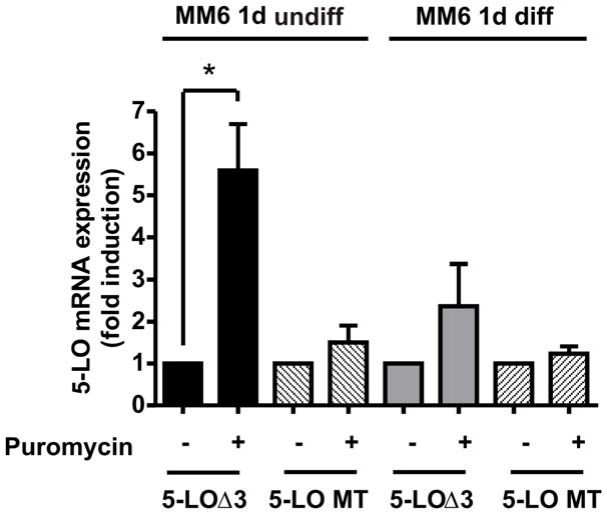
Effect of NMD inhibition by puromycin on 5-LOΔ3 splice variant and mature 5-LO transcript expression. The cells were cultured with and without TGFβ (1 ng/ml) and calcitriol (50 nM) for 24 h. Differentiated and undifferentiated MM6 cells were cultured for 24 h and finally treated with puromycin (300 µg/mL) for 8 h. Total RNA was isolated and the cDNA was prepared by random hexamer priming. The cDNA was amplified by PCR using primer pair G for the analysis of 5-LOΔ3 mRNA. For RT-PCR analysis of the mature 5-LO transcript (5-LO MT), the total RNA was reverse transcribed using oligo-dT primers. PCR was performed using primer pair F and β-actin mRNA served as constitutively expressed control. Variations in the expression levels compared to non-treated samples (set as 1) are given as the mean+SE of three or four independent experiments, *p<0.05.

To analyze this, we exposed 1 d differentiated and undifferentiated MM6 cells to the protein synthesis inhibitor puromycin. Puromycin leads to a strong accumulation of the alternative transcript in undifferentiated cells (Fig. 3). Interestingly, 5-LOΔ3 was only slightly affected by puromycin in differentiated cells. The mature 5-LO transcript was insensitive to puromycin in differentiated as well as undifferentiated cells.

**Figure 4:**
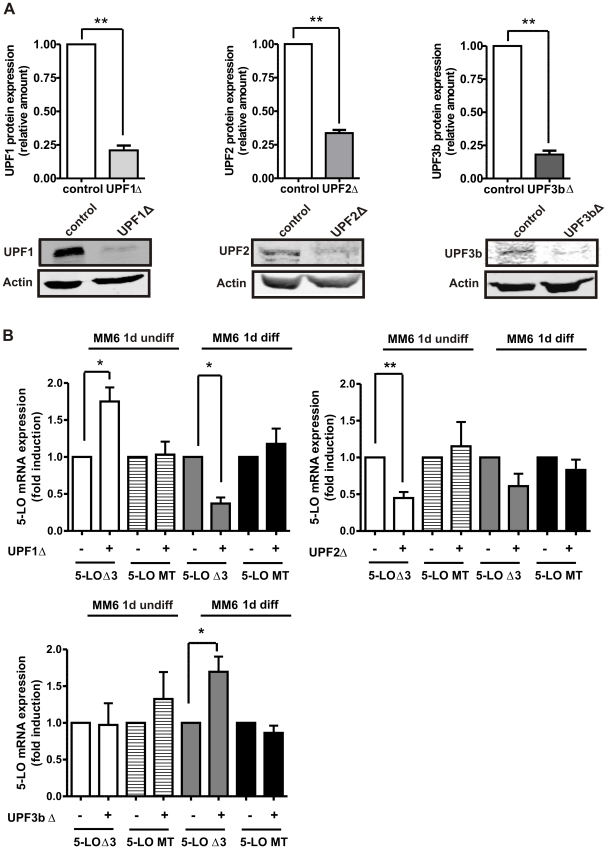
Individual knockdown of NMD factors in MM6 cells. (A) Western blot analysis of UPF1, UPF2 and UPF3b protein expression in MM6 cells with and without knockdown of UPF1, UPF2 and UPF3b, respectively. Cell lysates (50–250 µg protein) in SDS-PAGE loading buffer (5×) were separated by 10% SDS-PAGE and transferred to a nitrocellulose membrane by electro blotting. The membrane was incubated with UPF1, UPF2, UPF3b and β-actin antibody. Visualization of protein bands was carried out by the use of infrared dye-conjugated antibodies (IRDye®), and analysis was performed with the Odyssey® infrared imaging system (*LI-COR*® Biosciences). β-actin was used as loading control. The relative changes of UPF knockdown samples to control samples (set as 1) are given as mean+SEM of three independent experiments, **p<0.01. (B) Effect of UPF knockdowns on 5-LOΔ3 and mature 5-LO mRNA expression in MM6 cells. The cells were cultured with and without TGFβ (1 ng/ml) and calcitriol (50 nM) for 24 h. Then, total RNA was isolated and cDNA was prepared by random hexamer priming. PCR analysis was carried out using primer pair G for 5-LOΔ3. For analysis of the mature 5-LO transcript, the total RNA was reverse transcribed using oligo-dT primers. PCR was performed using primer pair F and β-actin mRNA served as constitutively expressed control. Variations in the expression levels compared to non-treated samples (set as 1) are given as the mean+SE of three or four independent experiments, *p<0.05, **p<0.01.

To prove whether 5-LOΔ3 was degraded by NMD, we knocked down UPF1 by shRNA. UPF1 is an essential component of the NMD machinery and its downregulation usually leads to an upregulation of mRNAs which are targeted by NMD. The lentiviral expression of a UPF1-specific shRNA resulted in a 79% knockdown of UPF1 protein expression (Fig. 4A). The UPF1 knockdown resulted in a 2-fold increase in 5-LOΔ3 mRNA in undifferentiated MM6 cells (Fig. 4B). In contrast, a significant reduction of 5-LOΔ3 mRNA expression to ∼30% was observed in differentiated MM6 cells. As expected, the mature 5-LO transcript was insensitive to UPF1 knockdown in undifferentiated as well as in differentiated MM6 cells. The findings that 5-LOΔ3 mRNA is slightly increased by puromycin treatment in differentiated MM6 cells and is downregulated upon UPF1 knockdown raises some mechanistic questions since UPF1 is supposed to be a key component in the NMD complex. To gain further insights into the NMD of 5-LO mRNA in MM6 cells, we preformed knockdowns of the NMD factors UPF2 and UPF3b.

The knockdown of UPF2 and UPF3b lead to a 66% and 88% reduction of UPF2 and UPF3b protein expression, respectively (Fig. 4A). UPF2 knockdown resulted in a 55% decrease in 5-LOΔ3 mRNA expression in undifferentiated MM6 cells. In contrast, only a slight reduction of 5-LOΔ3 mRNA was observed in differentiated cells (Fig. 4B). Opposite effects on 5-LOΔ3 mRNA expression were detected when UPF3b was knocked down. Here, 5-LOΔ3 mRNA expression was not affected upon UPF3b knockdown in undifferentiated MM6 cells, but a significant upregulation of 5-LOΔ3 transcript expression was obtained in differentiated cells.

**Figure 5:**
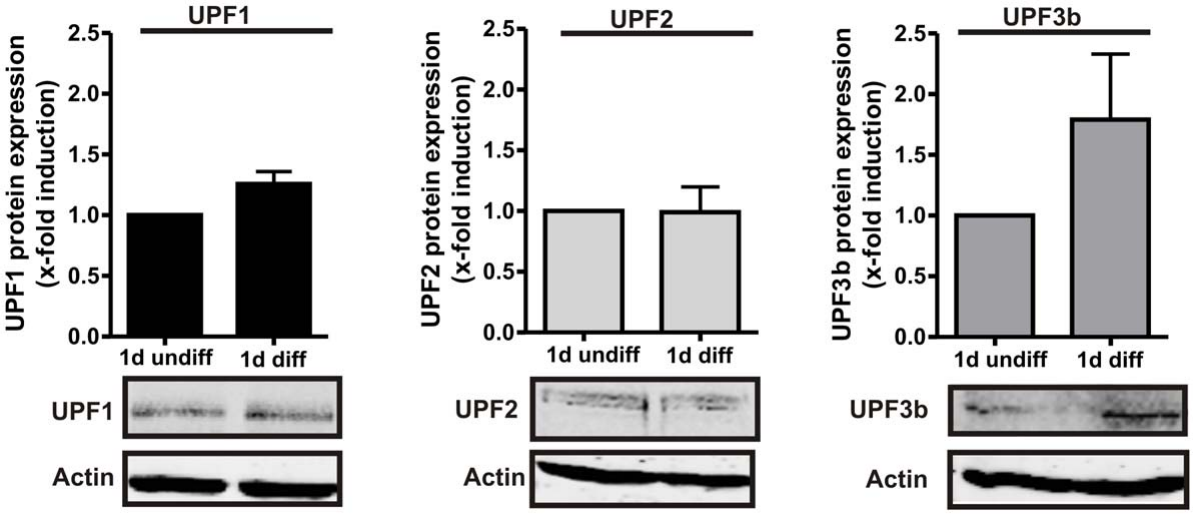
Western blot analysis of UPF protein expression in MM6 cells. Western blot analysis of UPF protein expression in MM6 cells incubated with and without TGFβ (1 ng/ml) and calcitriol (50 nM) for 24 h. Differentiation-dependent relative changes in UPF protein expression compared to undifferentiated samples (set to 1) are given as the mean+SE of three independent experiments.

Western blot analysis of UPF proteins was performed in order to investigate whether cell differentiation affects the expression of the UPF proteins. No significant changes in UPF1 and UPF2 expression were observed during differentiation of the MM6 cells (Fig. 5). Interestingly, an about 2-fold increase in UPF3b expression was detected when the cells were differentiated with calcitriol and TGFβ.

## Discussion

Alternative splicing of 5-LO transcripts was observed when 5-LO mRNA expression was investigated in human brain tumors [8]. Recently, the existence of alternatively spliced isoforms of 5-LO mRNA has been described in different cell lines and in leukocytes [9]. Here, a comprehensive analysis of the 5-LO splicing pattern demonstrated the existence of at least seven alternatively spliced transcripts in MM6 cells. Interestingly, induction of immature and/or alternatively spliced 5-LO transcripts preceded the increase in mature 5-LO mRNA following differentiation of MM6 cells by calcitriol and TGFβ. This is agreement with previous analyses where the induction of immature 5-LO transcripts preceded the upregulation of mature 5-LO mRNA by both agents. We could recently show that induction of 5-LO mRNA expression by calcitriol and TGFβ is mainly due to the stimulation of transcript elongation [7]. Since correctly and alternatively spliced immature 5-LO transcripts are upregulated with similar time courses it seems that both agents do not affect the splicing process. Interestingly, upregulation of mature 5-LO mRNA is significantly slower than the induction of the immature 5-LO mRNA species. One possible explanation is that alternatively spliced transcripts containing PTC could be subjected to NMD before transcript maturation so that only the regular transcripts accumulate after maturation. Thus, accumulation of the mature 5-LO mRNA and degradation of the other transcripts may be an explanation why only the mature 5-LO mRNA is detected in differentiated leukocytes by Northern blot [16]. Based on the “50–55 nucleotides”- rule [15] all new 5-LO splice variants reported here possess PTCs and may represent NMD targets. To confirm the NMD hypothesis, the most prominently expressed splice variant 5-LOΔ3 was characterized in this study. Since NMD depends on mRNA translation, MM6 cells were treated with the protein biosynthesis inhibitor puromycin. 5-LOΔ3 mRNA expression was significantly upregulated by puromycin. Interestingly, the puromycin effect depended on the differentiation status and an only slight increase in 5-LOΔ3 mRNA expression was found in differentiated MM6 cells. Differentiation-dependent effects on 5-LOΔ3 mRNA expression were also observed when NMD was inhibited by knockdown of UPFs. UPF1 knockdown caused a significant induction of 5-LOΔ3 mRNA expression in undifferentiated MM6 cells whereas a significant decrease was observed in differentiated MM6 cells (Fig. 4B). UPF2 knockdown resulted in a significant downregulation of 5-LOΔ3 mRNA expression in undifferentiated cells as well as to a weaker extend in differentiated MM6 cells (Fig. 4B). UPF3b knockdown did not affect 5-LOΔ3 mRNA expression in undifferentiated MM6 but lead to a significant upregulation in differentiated cells (Fig. 4B). All the data suggest that 5-LOΔ3 mRNA expression is regulated by NMD. According to the general model, one would expect that knockdown of UPF proteins inhibits NMD and leads to the upregulation of the transcript. One possible explanation for our observations that UPF knockdown leads to both up- and downregulation of 5-LOΔ3 mRNA expression is that there are several alternative NMD pathways that are dependent on cell differentiation. However, these cell differentiation-dependent changes do not seem to be due to alterations in the expression of the UPF proteins since we observed no change (UPF1, UPF2) or an only up to 2-fold upregulation of UPF3b protein expression after cell differentiation.

Alternative pathways of NMD have previously been proposed to exist in mammalians, but the role of these alternative NMD mechanisms has not been defined [17], [18], [19]. Gong *et al.* have shown that differentiation of C2C12 myoblasts to myotubes is accompanied with an increase in the efficiency of an alternative NMD and a decreased efficiency of classical NMD [20]. Recent studies suggest the existence of three different branches of NMD in mammals. The first is independent of UPF2, it retains its normal function when UPF2 is depleted [18]. In contrast, the second branch is independent of the core proteins of the exon-junction complex (EJC), but depends on the RNA-binding EJC protein RNPS1 and UPF2 [18]. And finally, the third alternative NMD mechanism is described as UPF3 independent [17].

Due to the different efficiencies of the components of the NMD complex to mediate mRNA degradation and the dynamics in the composition of the complex, it is not so surprising that the knockdown of single components of the NMD-EJC leads to up- as well as downregulation of the target mRNAs. Indeed, in a genome-wide analysis of NMD targets, Wittman et al. identified many genes that are differentially regulated by UPF1 and UPF2 knockdown in HeLa cells. Their findings suggest that UPF1 and UPF2 specific NMD could exist in mammalian cells [21].

Also, cell differentiation-dependent changes in NMD were observed with UPF2. It was found that NMD in differentiated cells were only mildly affected in UPF2 null mice, suggesting that UPF2-dependent NMD mainly occurs in proliferating cells like undifferentiated MM6 [22].

In contrast to 5-LOΔ3, the mature 5-LO transcript encoding the 5-LO protein does not contain a PTC and should not be subjected to NMD. In agreement with this, expression of 5-LO mRNA was not affected by puromycin treatment or by UPF knockdown in differentiated as well as in undifferentiated MM6 cells. This is in line with previous data, where we found that the protein biosynthesis inhibitor cycloheximide (which can also impair NMD) does not alter 5-LO mRNA expression in MM6 cells [23]. Additionally, the mature 5-LO transcript was insensitive to all UPF knockdowns in differentiated as well as undifferentiated cells.

Taken together, we identified a series of novel 5-LO splice variants in MM6 cells. Characterization of one of the most abundant of these 5-LO splice variants, 5-LOΔ3, showed that it is a NMD target. Our data suggest a model for post-transcriptional regulation of 5-LO gene expression by coupling of nonsense mediated mRNA decay and alternative splicing.

## Materials and Methods

### Cell culture

MM6 cells were obtained from DSMZ (DMSZ no. ACC124). MM6 cells were grown in RPMI-1640 medium supplemented with 10% (v/v) fetal calf serum (FCS, Biochrom AG), 100 µg/ml streptomycin (PAA The Cell Culture Company), 100 U/ml penicillin (PAA The Cell Culture Company), 1× non essential amino acids (Sigma Aldrich), 10 µg/ml insulin, 1 mM oxalacetate (AppliChem) and 1 mM sodium pyruvate (PAA The Cell Culture Company). Cell culture was carried out in a humidified atmosphere of 5% CO_2_ at 37°C. MM6 cells were differentiated with 1 ng/ml TGFβ and 50 nM calcitriol at 37°C, 6% CO_2_.

### RNA extraction and RT-PCR

Total RNA was extracted from 3 million MM6 cells with TRIzol Reagent (Invitrogen) and treated with Turbo DNase (Ambion) according to the manufacturer's instructions. 1.5 µg of DNase-treated RNA was used for first-strand cDNA synthesis using random hexamer primer (Fermentas) and 200 U SuperScript II reverse transcriptase (Invitrogen) in a reaction volume of 20 µl following manufacturer's instructions. The RNA was digested with 5 U RNAseH (New England Biolabs). The first-strand cDNA was finally used as template for PCR reactions with gene specific primers. PCR was carried out using 2.5 U Taq polymerase (New England Biolabs) according to the manufacturer's instructions with the addition of 50 µM betaine (Sigma Aldrich).

1 µg total RNA was reverse transcribed using the iScript™ cDNA Synthesis Kit (Bio-Rad) to analyze mature 5-LO transcripts. The first-strand cDNA solution was used for PCR using 2.5 U Taq polymerase (New England Biolabs) according to the manufacturer's instructions with the addition of 50 µM betaine (Sigma Aldrich). The primers Exon13F and 3′UTR-R were used for specific amplification of mature 5-LO mRNA (according to [23]). All primer sequences are listed in the Table S1.

Amplification of the different PCR products was preformed as follows: each cycle consisted of denaturing at 95°C for 45 s, depending on the primer pair the annealing temperature was between 53 and 58°C for 60 s, and primer extension at 72°C for 45 s. The annealing temperature was individually adjusted. Cycle numbers were minimized for each series of experiments in order to keep the PCR reaction in the exponential phase and to avoid saturation effects (5-LO specific primer: 30–35 cycles; actin 25 cycles).

One tenth of each PCR sample was analyzed by gel electrophoresis in a 1.5% ethidiumbromide-stained agarose gel. Images of the gels were digitally captured by an image analysis system and the BIO-ID software (Vilber Lourmat).

### 5′ Rapid amplification of cDNA ends (5′RACE)

12 µg DNase-treated total RNA was treated with 2 U alkaline phosphatase (Roche Applied Science) for 1 h at 37°C according to the manufacturer's instructions. The RNA was purified by phenol/chloroform/isoamyl alcohol (Roti® Phenol/Chloroform/Isoamyl alcohol, Carl Roth), then treated with 30 U tobacco acid pyrophosphatase (TAP, Epicentre Biotechnologies) for 90 min in the supplied buffer and precipitated. TAP treatment was omitted for a negative control. The sample was mixed with 400 pmol of a 40 nt RNA linker (Table S1). RNA ligation was performed in a reaction volume of 20 µl using 5 U T4 RNA ligase 1 (New England Biolabs) or 5 U T4 RNA ligase (Ambion) for 90 min in the supplied buffer. 10 µl ligation product was reverse transcribed using random hexamer primers (Fermentas), 200 U SuperScript II reverse transcriptase (Invitrogen) and afterwards treated with 5 U RNAse H (New England Biolabs). The cDNA library was used as template for nested PCR with the linker specific and gene specific primers (Table S1).

### Cloning of PCR and 5′RACE products

QIAquick Gel Extraction Kit (Qiagen) was used for gel extraction of the RT- and 5′RACE products. The isolated PCR fragments were cloned into the pDrive cloning vector using the Qiagen PCR Cloning^PLUS^ Kit (Qiagen) which allows blue/white screening of recombinant colonies. The selected recombinant plasmids were then sequenced.

### Western blotting

Cells were lysed in phosphate buffered saline (PBS, PAA The Cell Culture Company), 0.1% Triton X-100 (Fluka BioChemika), 1 mM phenyl methane sulfonyl fluoride (PMSF, Fluka BioChemika) and centrifuged for 10 min (10,000 g, 4°C). The protein content of the supernatant was determined by Bradford colorimetric assay [24] (BioRad Laboratories). 50–250 µg protein lysate was separated by SDS-PAGE (10%), transferred to a HyBond ECL nitrocellulose membrane (Amersham) and blocked with Odyssey blocking buffer (*LI-COR*® Bioscience) for 1 h at room temperature. The membranes were then incubated over night at 4°C with primary antibodies that recognize 5-LO (BD Bioscience), GAPDH (Cell Signaling), UPF1, UPF2, UPF3b (antiserum was generously supplied by Jens Lykke-Andersen, University of California San Diego) and β-actin (Santa Cruz Biotechnology). Membranes were washed with PBS pH 7.4/Tween20 0.1% (v/v) (Carl Roth) and incubated with infrared dye conjugated secondary andibodies (IRDye®, *LI-COR*® Bioscience) for 45 min at room temperature. Visualization and quantitative analysis was carried out with the Odyssey Infrared Imaging System (*LI-COR*® Biosciences).

### NMD inhibition by puromycin

MM6 cells were cultured with and without TGFβ (1 ng/ml) and calcitriol (50 nM) for 24 h. Differentiated and undifferentiated MM6 cells were cultured for 24 h and finally treated with puromycin (300 µg/mL, Alexis Corporation) for 8 h as described [25].

### shRNA-mediated knockdown of UPF factors in MM6 cells

A lentiviral expression system for shRNA expression was used for UPF1 knockdown. The MISSION shRNA plasmid for UPF1 knockdown (NM_002911.2-2451s1c1, TRCN0000022257), for UPF2 knockdown (NM_015542.2-1035s1c1, TRCN0000151381) and for UPF3b knockdown (NM_023010.2-1007s1c1, TRCN0000152769) was obtained from Sigma Aldrich. For the generation of lentiviral particles HEK293T cells were transfected with 10 µg of the shRNA plasmid and 10 µg packaging plasmids (VSVG) by the calcium phosphate method [26]. The medium was changed after 4 h and cells were cultured for 72 h. For transduction, MM6 cells were supplemented with protamine sulphate (4 µg/ml, Calbiochem). 500 µl of the viral supernatant were added to cell suspension and centrifuged (2500 rpm, 90 min, 32°C). The transduced cells were grown for two days before puromycin was added (0.75 µg/ml) to select the transduced clones. After three weeks, knockdown efficiency of UPF factors was analyzed by Western blot.

### Statistics

Results are given as mean+SEM of three or four independent experiments. Statistical analysis was carried out by Student's paired t test (two-tailed) using GraphPad Prism 5.0. Differences were considered as significant for p<0.05 (indicated as * for p<0.05, and ** for p<0.01).

## Supporting Information

Figure S1 **Location of the primer pairs used for the 5′RACE and RT-PCR analysis (indicated with letters A to G) on the 5-LO cDNA.**
(TIF)

Table S1 **Primer sequences.**
(DOC)
