# Supplementary figures and images for: Post-Transcriptional Regulation of 5-Lipoxygenase mRNA Expression via Alternative Splicing and Nonsense-Mediated mRNA Decay
Source: PLoS One. 2012 Feb 21;7(2):e31363. doi: 10.1371/journal.pone.0031363 (PMC3283622; doi:10.1371/journal.pone.0031363)

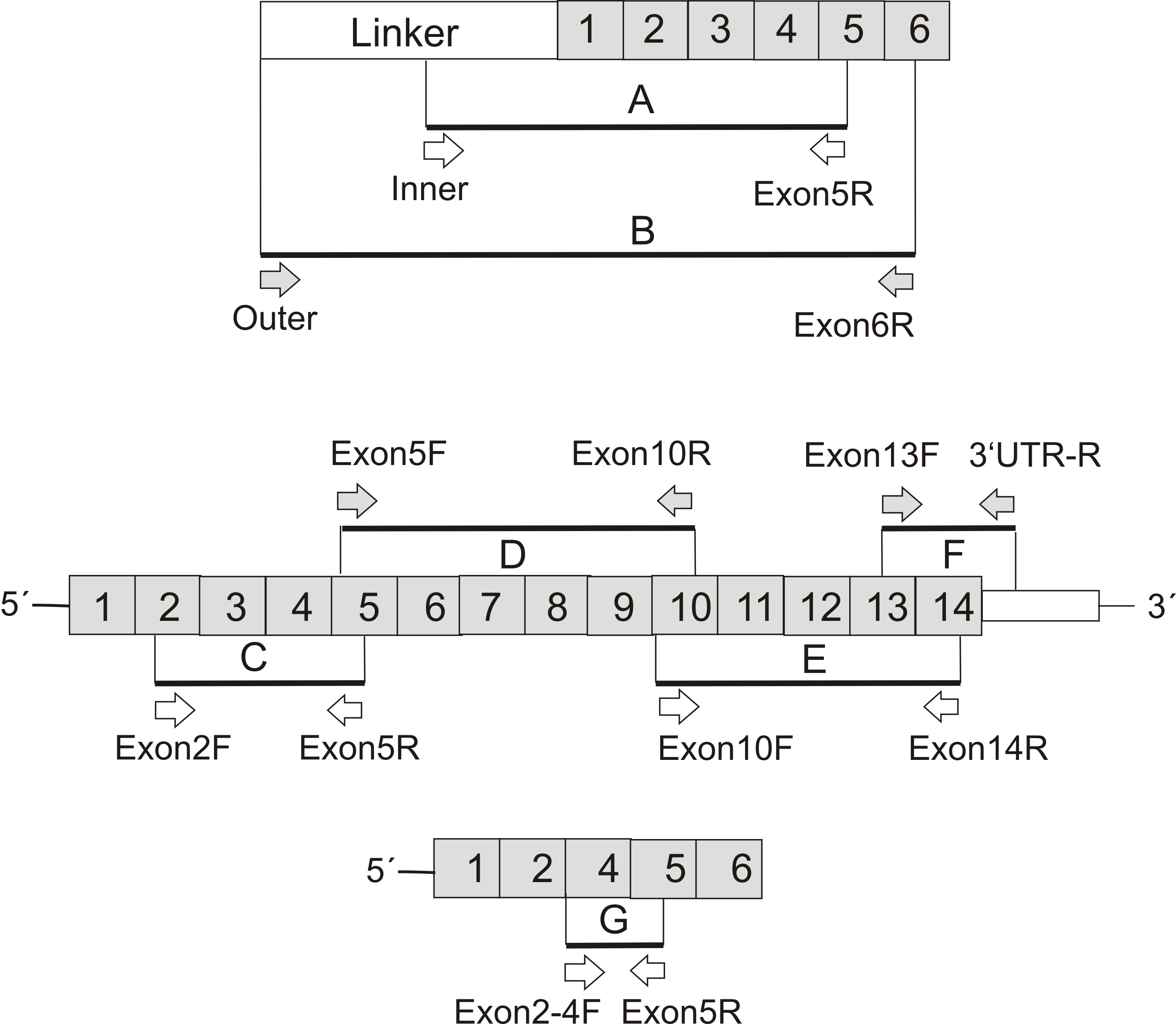

Supplement: Figure S1 — Location of the primer pairs used for the 5′RACE and RT-PCR analysis (indicated with letters A to G) on the 5-LO cDNA. (TIF) [file pone.0031363.s001.tif]
