# Supplementary material for: Post-Transcriptional Regulation of 5-Lipoxygenase mRNA Expression via Alternative Splicing and Nonsense-Mediated mRNA Decay
Source: PLoS One. 2012 Feb 21;7(2):e31363. doi: 10.1371/journal.pone.0031363 (PMC3283622; doi:10.1371/journal.pone.0031363)
Supplement: Table S1 — Primer sequences. (DOC) [file pone.0031363.s002.doc]

**Table S1.** **Primer sequences.**

| Primer | Sequence |
| --- | --- |
| Exon2F | CATACGACGTGACTGTGGACGAGG |
| Exon2-4F | ATGGACGCGATGGATGGAG |
| Exon5F | CCTGTTCATCAACCGCTTCATGC |
| Exon5R | GTCGGCGAAGTCATTCCAAGAAG |
| Exon6R | GGACCTCCTGCTCCAAGCTGAG |
| Exon10F | GCATGGAGAGCAAAGAAGACATCC |
| Exon10R | CCCGTCGTCCCGGTAGAAGTAGTAG |
| Exon13F | TACGACTGGTGCTCCTGGATC |
| Exon14R | GGTCTGGGGACAAGTAGTAATATGGCAG |
| 3’UTR-R | GCAGTCCTGCTCTGTGTAGAATGGG |
|  |  |
| ActinF | CGGGACCTGACTGACTACCTC |
| ActinR | CTTCTCCTTAATGTCACGCACG |
|  |  |
| Inner | GGACACTGACATGGAGGAGGGAGTA |
| Outer | GACGAGCACGAGGACACTGACA |
|  |  |
| *RNA:* |  |
| Linker | GACGAGCACGAGGACACUGACAUGGAGGAGGGAGUAGAA |
